# Sensorimotor Self-organization via Circular-Reactions

**DOI:** 10.3389/fnbot.2021.658450

**Published:** 2021-12-13

**Authors:** Dongcheng He, Haluk Ogmen

**Affiliations:** Laboratory of Perceptual and Cognitive Dynamics, Department of Electrical & Computer Engineering, Ritchie School of Engineering and Computer Science, University of Denver, Denver, CO, United States

**Keywords:** sensorimotor learning, cognitive modeling, developmental robotics, perception-action coupling, reaching

## Abstract

Newborns demonstrate innate abilities in coordinating their sensory and motor systems through reflexes. One notable characteristic is circular reactions consisting of self-generated motor actions that lead to correlated sensory and motor activities. This paper describes a model for goal-directed reaching based on circular reactions and exocentric reference-frames. The model is built using physiologically plausible visual processing modules and arm-control neural networks. The model incorporates map representations with ego- and exo-centric reference frames for sensory inputs, vector representations for motor systems, as well as local associative learning that result from arm explorations. The integration of these modules is simulated and tested in a three-dimensional spatial environment using Unity3D. The results show that, through self-generated activities, the model self-organizes to generate accurate arm movements that are tolerant with respect to various sources of noise.

## 1. Introduction

Reaching for a desired target in space is a fundamental human sensorimotor ability. Many processes are involved in this ability, including stereovision to recover the position of the target in the three-dimensional space, motor control to move the arm, and visuospatial sensorimotor learning to coordinate sensory and motor representations (Mackrous and Proteau, 2016). Goal-directed reaching involves the detection and recognition of the object of interest among surrounding objects in space, determining its spatial position, and finally guiding the arm toward that position.

Goal-directed reaching has many applications in robotics and has been approached both from the perspective of physical modeling (forward and inverse arm kinematics) (Goldenberg et al., 1985; Manocha and Canny, 1994; Parikh and Lam, 2005; Mohammed and Sunar, 2015; Srisuk et al., 2017; Reiter et al., 2018) as well as from the perspective of biological-system modeling. Physical modeling approaches rely heavily on the accurate and explicit model of the arm (length of limbs, etc) and require re-calibration when physical parameters undergo unforeseen changes. On the other hand, biological systems exhibit remarkable adaptability; for example, the size of a growing child's arm changes but the brain can adapt and “automatically re-calibrate” its sensorimotor control processes. This is one reason why several researchers focused on biologically-based approaches to sensorimotor control (Saxon and Mukerjee, 1990; Asuni et al., 2003, 2006; Laschi et al., 2008; Hoffmann et al., 2017).

Approaches to biologically-based sensorimotor control are influenced by psychological theories of intelligence. According to behaviorism, the motor system produces observable behaviors which constitute the fundamental level of analysis (Graham, 2000; Sherwood and Lee, 2003). Strict behaviorism proposes that the analysis of intelligence should be based solely on observable variables, viz., stimuli and responses (behavior), without any reference to the system itself. In other words, the biological system is treated as a “black box,” and learning is defined as changes in behavior as a result of two associative processes: In classical, or Pavlovian, conditioning, changes in behavior result from associating one stimulus (conditioned stimulus, CS) with another one (unconditioned stimulus, US), which is contingent on CS. In instrumental or operant conditioning, changes in behavior occur as a result of a reinforcing stimulus which is contingent on the behavior produced by the organism. This approach is exemplified with the currently popular deep-learning models that use a dataset containing inputs (stimuli) and desired outputs (reinforcement signals) and train a multi-layer network whose architecture is defined mostly in an *ad-hoc* manner. In contrast, constructivist theories put a central role on the internal processes of the organism that actively structures its inputs (Piaget, 1952). Hence, constructivist approaches place a central role for structural and functional properties of the organism. Our approach follows this latter theoretical perspective by incorporating modules that are inspired from the structure, i.e., functional neuro-anatomy of the primate brain, and the functional principle of “circular reactions” (Piaget, 1952).

The primate cortex consists of two general pathways: the dorsal and ventral pathways (Goodale and Milner, 1992). These two pathways carry out complementary information processing: The ventral pathway is specialized for processing “what” information, i.e., the detection and recognition of objects. The dorsal pathway is specialized for the “where” information, i.e., the localization of objects in space. From its definition, it is clear that goal-directed reaching necessitates both the ventral (detection and recognition of the desired target) and the dorsal pathways (localization of the desired target in order to guide arm movements). The joint operation of these two pathways suggests interactions between them. Indeed, neurophysiological findings suggest that the “what” and “where” specializations are not binary exclusive properties of these pathways but are shared to some extent (Mishkin et al., 1983; Wang et al., 1999; Sereno et al., 2014). Neurophysiological studies also indicate heavy connectivity between these areas, possibly underlying their joint synergetic operations (Rosa et al., 2009; Wang et al., 2012; Van Polanen and Davare, 2015). In our model, we start with egocentric visual representations that reflect the coding of visual information in early visual areas of the cortex. Through the optics of the eye, neighboring points in the environment are projected to neighboring points on our retinas. These neighborhood relationships are preserved by retino-cortical projections. This organization is called retinotopic organization (Engel et al., 1997). The retinotopic cortical areas constitute a map representation in the sense that the location of active neurons indicates the location of the stimulus with respect to the positions of the eyes. The eye-based representation is an egocentric map because the location is encoded with respect to the eyes of the observer. An egocentric reference frame is one that is relative to the subject, e.g., eye-, head-, body-, limb-based reference-frames. In the next stage of the model, disparity information is used to combine the two egocentric retinotopic maps into an exocentric “cyclopean map.” Exocentric reference-frames are those that are relative to a reference outside the subject^1^. For example, in the cyclopean map, the position of an object is with respect to its position in the external world and hence its coded position does not change when the eyes move. This exocentric representation is then coordinated with motor representations by using the functional principle of circular reactions. Newborns start with genetically encoded reflexes, which consist of actions like sucking. These reflexive motor behaviors form circular reactions in that their end point becomes the beginning of a new cycle and this closed cycle repeating itself for autonomous learning and self-organization. These circular reactions allow the coordination of different senses and motor actions to guide the movements of our body (Piaget, 1952). Beginning with reflexes or random body explorations and repeating these procedures circularly, sensory and motor representations are gradually coordinated.

To model and simulate this sensorimotor self-organization, we propose and test an integrative model that combines several neural-network modules that are based on neuro-anatomical and functional properties of the primate visual system.

## 2. Related Work

As discussed in the previous section, several studies use the physical modeling to characterize the known structure of the arm and joints and the application of forward and inverse kinematics can be used to determine and move the arm to a desired location (Goldenberg et al., 1985; Manocha and Canny, 1994; Parikh and Lam, 2005; Mohammed and Sunar, 2015; Srisuk et al., 2017; Reiter et al., 2018). Biologically motivated studies that follow the behavioristic approach do not use a model of the arm but “learn” its structure through stimulus-response training. Our approach follows the constructivist tradition and incorporates structural and functional properties of the system, in this case the primate brain. Hence the key elements of our approach are egocentric and exocentric maps, motor vector representations, local associative coordination of maps and vectors through circular reactions.

Many studies indicated that in human, the developmental functions of brain is modulated by the sensory-motor experience (Barsalou, 2008; Schillaci et al., 2016). This skill is thought to be acquired through the active interactions with the external environment (Piaget, 1952). A typical scenario of this is the reaching behavior under the guidance of visual information, which has been widely simulated by various of modeling structures. For instance, Saxon and Mukerjee (1990) studied sensory-motor coordination by self-organizing neural networks, and created associations between an egocentric visual map and a motor map via circular reactions. The problem was simplified into a two-dimensional working space and was simulated with a robotic arm consisting of three degrees of freedom. Another study, described in Asuni et al. (2003, 2006), offered a more developed system working in three-dimensional space and simulated with a DEXTER robotic arm. But the visual space was effectively two-dimensional since the target objects were located on a planar table. Similarly, the model learned through circular reactions and the motor system was represented by vectors. However, the objects visual locations were represented by gaze positions (vectors).

Some studies focused more on the learning mechanisms. For instance, Santucci et al. (2014) proposed a model incorporating a novel reward mechanism that used the dopaminergic neurons to strengthen the learning effect of reaching behaviors. This model was simulated on a robotic arm, which moved in a three-dimensional space, even though it was tested with target objects located on a table. The neural networks were trained via reinforcement learning where both the visual inputs and the motor system were represented by vectors. Tanneberg et al. (2019) implemented a stochastic recurrent network to refine the end-effector's motion trajectory to avoid the obstacles on the way during reaching. In their study, visual inputs were not used. In some studies more complex hand movement scenarios, like grasping were implemented (Sarantopoulos and Doulgeri, 2018; Della Santina et al., 2019).

In recent years, the scope of this research area has been expanded to a larger variety of tasks beyond vision-based reaching. For instance, Hoffmann et al. (2017) implemented reaching with tactile stimuli and incorporated a transformation between the tactile map and motor coordinates. The robot learned through self-generated random babbling and a self-touch. Laschi et al. (2008) incorporated a visual processing module that is able to predict the object's tactile properties. The model learned the reaching direction and object orientation and mapped the arm and hand coordinations to the objects geometrical features so that it was able to predict a suitable movement to grasp the object. Chao et al. (2010) developed an ocular-motor coordination that gradually mapped the gaze space and the motor space of the ocular muscles. This study included the differential resolution found on the retina (fovea vs. periphery) and used eye movements (saccades) to bring the stimulus from the periphery to the fovea. Schmerling et al. (2015) used a robot with head motion and suggested that head-arm coordinations would improve learning. The neural network drove the goal-directed reaching of an arm of a robot and were trained through circular reactions. The objects positions were represented by head's rotation vectors and thus the study did not address how exocentric reference frames are produced. Pugach et al. (2019) proposed a “gain field” neural model where tactile information is included to establish a mapping between visual and motor spaces. Our model also uses gain field neurons and processes the motor commands through neural population encoding. This computational principle has been found to play an important role in goal-directed sensory-motor transformation (Andersen and Mountcastle, 1983; Salinas E, 2001; Pouget et al., 2002; Blohm and Crawford, 2009).

The novelty of the present model is that we provide a neurally plausible solution to the coordination between motor configurations and an exocentric map, which is generated and associated with joint vectors simultaneously. Currently, some approaches to build and expand the visual map are reported. For instance, Jamone et al. (2014) presented a strategy by which the robot learns to expand and associate the visual maps in different body positions by goal-directed reaching movements. However, the visual map in their study is not a map of neurons representing the spatial relationships among all the objects in the external environment. Instead, it is a map representing the reachability of each fixation point. The model was simulated on a robot in a three-dimensional space. Chao et al. (2016) proposed a robotic system utilizing a visual processing approach inspired from human retina. The method uses a head motor system to transform the spatial locations to the head joint vectors. In this method, spatial locations are reflected by the motor vectors instead of the inter-spatial relationships in the retinotopic map. Other studies reproducing this method also include (Shaw et al., 2012; Law et al., 2013). In contrast to those approaches, the exocentric reference frame in our model is built by fusing two retinotopic maps and by compensating for eye movements.

## 3. Description of the Model

**Figure 1:**
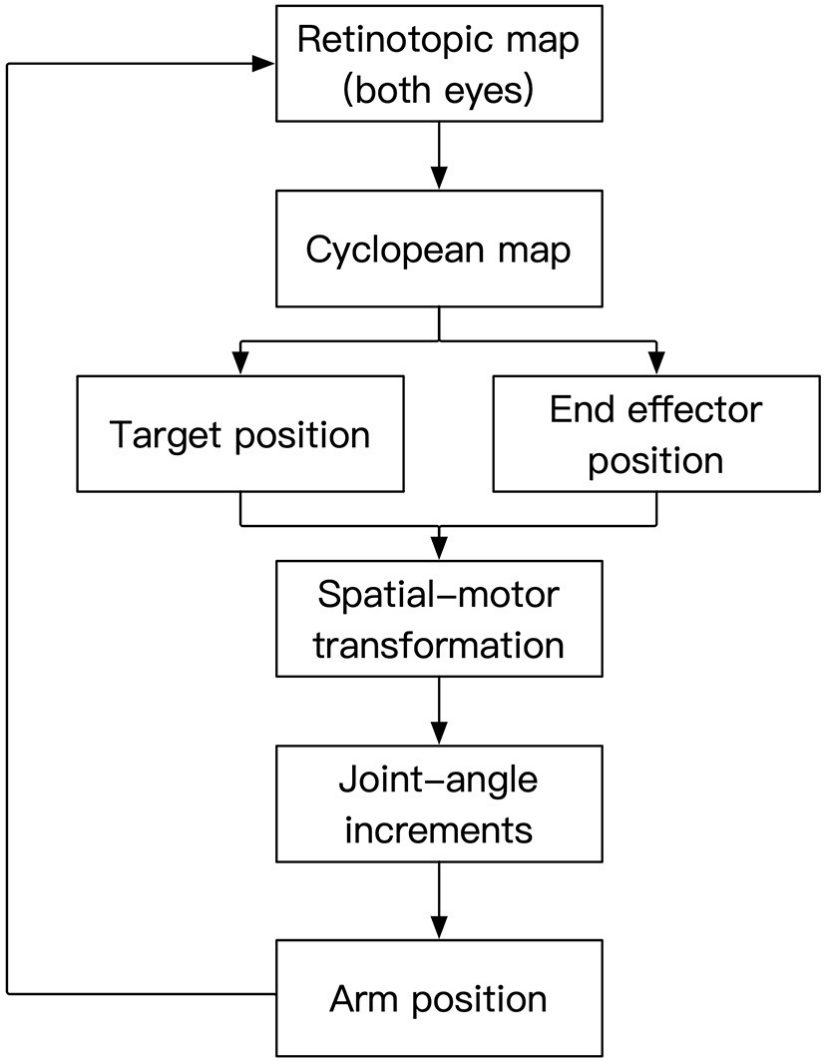
Processing stages of the model. Two-dimensional retinotopic maps from the left and right eyes are combined into a cyclopean map to reveal depth information. This information is used to represent target and end-effector positions in an exocentric reference-frame. The spatial position information is then converted to equivalent motor representations, which in turn drive the movements of the joints to have the end effector reach the target position. The visual input corresponding to the moving arm established a feedback loop to control the action.

The cortical organization reflects interactive functioning of many specialized modules, anatomically corresponding to various “areas” of the cortex. In a similar way, as shown in Figure 1, our model consists of interacting modules. In this study, for simplicity, we limited senses to vision and motor control to one arm. Our model receives the visual input through its two “eyes” and encodes this information retinotopically as in human early visual areas (Engel et al., 1997). In human vision, the environment is projected on the retina through the optics of the eyes following perspective geometry. Hence, neighboring points in the environment are imaged on neighboring retinotopic positions. These neighborhood relations are preserved through the precise connections from retina to early visual cortex giving rise to the “retinotopic organization” of early visual areas. Retinotopic areas provide an egocentric map representation for the stimulus. This is called a “map” representation (Bullock et al., 1993) because the relative position of each neuron with respect to its neighbors carries information about the position of the stimulus, much like the representation of cities on a map carries information about their relative locations. It is an egocentric map because the location information is relative not only to the position of the stimulus in space but also relative to the position of the eyes. The next step in the model is to recover the position of the stimulus in space in a way that is invariant with respect to the positions of the eyes, i.e., an exocentric representation. Hence, at the next stage, the two retinotopic maps are fused by using the binocular disparity information to build a “cyclopean map” (Julesz, 1971). The arm position is represented by neurons that encode joint angles in a vector format. In this “vector representation” (Bullock et al., 1993), each group of neurons is associated with a joint and the activities of these neurons encodes in an analog way the joint angle, e.g., the higher the activity the larger the joint angle. Synaptic connections between sensory map-representations and motor vector-representations allow the coordination of these activities through circular reactions. To initiate the circular reaction, we send a random command to joint angles which then moves the arm accordingly. As the arm moves, its image is represented in the retinotopic maps, creating a visuo-motor feedback loop. Through this self-generated action, the system activates motor and sensory representations and these simultaneous activities provide the input for associating sensory and motor activities that are congruent with the physics of the external world. This way, we do not need to incorporate physical models of the arm, eyes, etc., the system learns the relationships of the joints, limbs, eyes, etc by perceiving the consequences of self-generated actions. An important implication of this type of learning is that the system does not need explicit models, parameters but constantly adapts and recalibrates through action-perception-learning loops. Hence, self-organization, adaptation, and re-calibration are emergent properties of this approach. The synaptic plasticity of the connections between these sensory and motor representations coordinates them through associative learning. Once these coordinations are learned, a target position can predict the corresponding associated joint angles for the arm to reach the target and vice-versa.

### 3.1. Visual Processing: Retinotopic and Cyclopean Maps

A central feature of our model is that we process the egocentric (retinotopic) maps from the stereopair image to produce an internal visuomotor spatial representation-the cyclopean map (CPM) (Julesz, 1971), serving as an exocentric map to guide reaching activities. Our model implements this process in three steps: (1) coordinate the stereopair mapping; (2) compensate the binocular disparity and expand the depth dimension; (3) synthesize the luminance profile by a weighted sum of the luminance profiles of the stereopair after disparity-compensation.

#### 3.1.1. Coordination of Retinotopic Mappings

A stereopair consists of two retinotopic images that have mostly horizontally-shifted luminance profiles. This is because, given the horizontally displaced positioning of the two eyes, a point in the three-dimensional environment is projected onto horizontally-shifted locations in the left and right retina. This relative spatial displacement is called binocular disparity. Our model determines the binocular disparity information according to the criterion of spatial-frequency similarity. This process can be intuitively described as follows: The retinotopic maps are firstly demarcated into non-overlapping unit areas (UA) with uniform shapes. Thereafter, a spatial frequency filter that is slightly larger in size is applied to all the unit areas to obtain the response signals of each of them. After this step, a UA in one image can then be matched to another UA in the other image if they have the most related spatial frequency response across all areas. Importantly, the filter is larger than those unit areas, which means that the correspondence among unit areas are determined with considerations of not only the unit area itself but also its neighbors. In the following illustrations, we use (a,b) to indicate the size of neural maps, where “a” is the number of cells in rows and “b” in columns.

**Figure 2:**
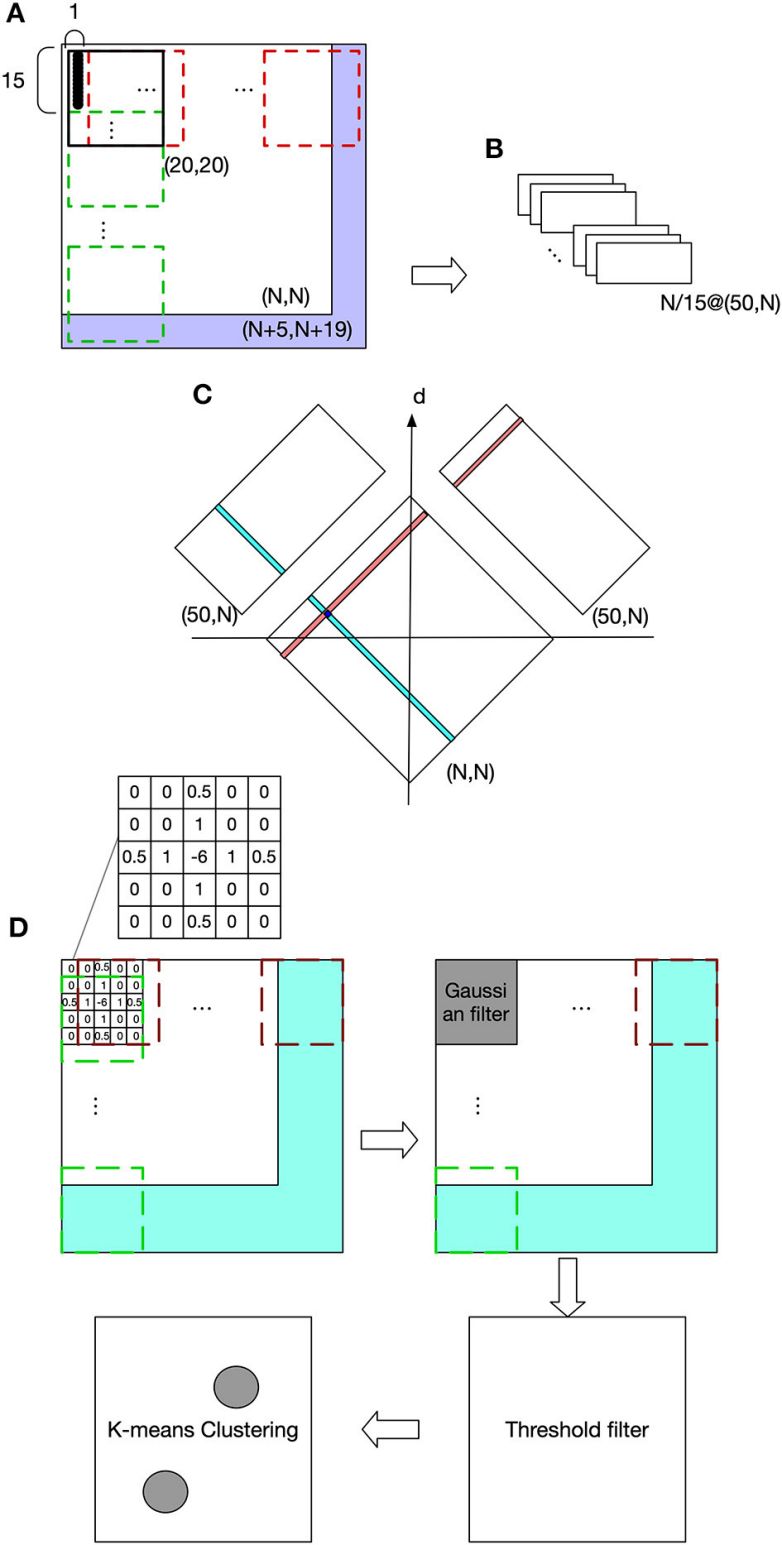
Visual processing. **(A)** The purple area shows zero pads. The red dashed frame indicates transformer's spatial extent along columns and the green dashed frame along rows. **(B)** Spatial frequency response maps (SfRM) with size of (50, N). Each row of UA is projected to a SfRM, within which each column is the frequency response of an UA. Therefore, a LumM possessing N/15 rows and N columns of UA creates N/15 SfRM in size of (50, N). A SfRM has 50 rows because 50 frequency responses are obtained, and N columns come from the corresponding N columns of UA. **(C)** Binocular correspondence map (BCM). Frequency-based comparisons determine the correspondence relations between the left and right UAs, and also their depth. **(D)** Spatial localization of the target object and the end-effector.

Figure 2 shows the visual processing beginning with a retinotopic map, with a size of (N, N), and it is firstly converted to a luminance map (LumM) by transforming the colored image to a gray scale image. This LumM was demarcated into N*N/15 UA with a size of (15,1). This UA can be thought as the resolution by which binocular disparity and depth are determined. Then spatial-frequency filters corresponding to multiple frequency-channels are applied to LumM. Here we used a two-dimensional Fast Fourier transformer with 50 frequency responses (from 1 to 50 cycles/deg). LumM was zero-padded to (N+5, N+19) and then scanned by a transformer in size of (20,20), which covered a UA and its surround. As a result, N/15 spatial-frequency response-maps (SfRM) of size (50, N) were obtained. N/15 Binocular Correspondence Maps (BCMs) in (N, N) were then generated using Equations (1) and (2), where *SfRM*_*l,i*_(*j, k*) represents *k cycles*/*deg* spatial-frequency response of an area registered by a UA in *ith* row and *jth* column of left retinal LumM (replace *l* by *r* to represent right retina). In Equation (1), the squared difference between the frequency-contents of the corresponding left- and right-eye patches are computed and its minimum provides the best matching binocular pair in terms of frequency contents. *BCM*_*i*_(*x, y*) was used to indicate the correspondent pairs in *ith* row of left and right retinal LumM. For a row *i* and each *x* from 1 to N,


(1) $$\begin{array}{l} y=\underset{j}{\min}\overset{50}{\underset{k=1}{\sum }}{\left(SfRM_{l,i}\left(x,k\right)-SfRM_{r,i}\left(j,k\right)\right)}^{2} \end{array}$$

(2) $$\begin{array}{l} \{\begin{array}{l} BCM_{i}\left(x,y\right)=1\\ BCM_{i}\left(x,ŷ\right)=0,\forall ŷ\notin y \end{array} \end{array}$$

According to Equations (1) and (2), each activate cell in a BCM encodes a binocular-correspondence relationship between a UA on left retinal LumM and another one on the right.

#### 3.1.2. Disparity Compensation and Depth Recovery

The depth of a UA can be determined by its representative neuron's relative position on the BCM map with respect to the d axis as shown in Figure 2C. In other words, on BCM, a UA's representative neuron's position along d will be equal to the depth of activated neurons when projected onto CPM. Since there are N placeholders along the depth dimension, the size of CPM is (N, N, N). This depth recovery approach follows Hirai and Fukushima's neural network model for extracting binocular parallax (Hirai and Fukushima, 1978). This process can alternatively be explained by the existence of a group of “binocular depth neurons” that are selectively sensitive to a binocular stimulation with a specific amount of parallax. Take the activated neuron in Figure 2C for example, this neuron in BCM becomes active only when it receives simultaneous stimulation of a UA whose position in the column is indicated by teal color and the UA marked by red. In fact, “binocular-depth neurons” have been found in many species including monkey and mouse (Poggio et al., 1988; La Chioma et al., 2020).

#### 3.1.3. Binocular Combination

Stereo image pair's luminance profiles are combined by summing them with a pair of physiologically plausible weights defined by a simplified version of Ding-Sperling model (Ding and Levi, 2017). This model was built based on the principle that each eye uses a gain-control on the other eye's signal in proportion to the contrast energy of its own input. After this procedure, the fused luminance profiles are then filtered and clustered based on the contrast change of the luminance. According to Ding-Sperling model, each spot of the luminance map is allocated with respect to the contrasts of that spot from the two eyes. This means that contrasts will be rebalanced toward the eye carrying the larger contrast energy. This contrast rebalancing is used, because simply taking the luminance distribution from single eye, without the contrast rebalance, weakens the effects of contrast-based clustering. It deteriorates the precision of cluster center localization when two objects are spatially closed. In this model, for each row, the total luminance *I*_0_ was determined by Equation (3), in which *x* is the number of the column, and *I* indicates the luminance. The contrast at *x*, *C*(*x*), is determined by Equation (4). Equation (5) provides the definition of the spatial-frequency filter *LoG*_*i*_(*x*). The contrast at *ith* spatial frequency *C*_*i*_(*x*) can be calculated by the convolution between *ith* spatial frequency filter and luminance profile of this row. The contrast energy in the *ith* channel, ϕ_*i*_, can then be calculated by Equation (6). The total contrast energy Φ is defined as the sum of contrast energies for all spatial frequency channels. And the total luminance energy L is determined by Equation (7). Φ_*L*_ and $L_{L}$ are the total contrast and luminance energies for a row in a left retinal UA, and energies for right retinal UA are given by Φ_*R*_ and$L_{R}$.The weights *w*_*L*_ and *w*_*R*_, which are used to combine a left retinal UA and its paired right UA, are calculated using Equation (8). The luminance profile of CPM is then determined by summing all UAs on the left LumM with their corresponding UAs on the right LumM according to these weights.

The above processes can be abstracted and summarized as follows: A UA on the left retina is firstly matched with a corresponding UA on the right retina based on the similarity of their spatial-frequency contents. These two UAs are assumed to be projected from the same environmental stimulus. These two UAs of size (15,1) are then projected to 15 neurons on the CPM, where their relative locations indicate their spatial position in the environment. The output of these 15 neurons on CPM are determined by weighted sum of two UAs' luminance profiles.


(3) $$\begin{array}{l} I_{0}=\underset{x}{\sum }I\left(x\right) \end{array}$$

(4) $$\begin{array}{l} C\left(x\right)=\frac{I\left(x+2\right)+I\left(x\right)-2I\left(x+1\right)}{I_{0}} \end{array}$$

(5) $$\begin{array}{l} \begin{array}{c} LoG_{i}\left(x\right)=-\frac{1}{\pi \sigma_{i}^{2}}\left(1-\frac{x^{2}}{\sigma_{i}^{2}}\right),\\ \sigma_{i}=5*i,i=1,2,3,...,10 \end{array} \end{array}$$

(6) $$\begin{array}{l} \{\begin{array}{l} C_{i}\left(x\right)=\frac{1}{I_{0}}LoG_{i}*I\left(x\right)\\ \phi_{i}=\sum_{x}\left(w_{c}\left(x\right)\cdot C_{i}\left(x\right)\right),w_{c}=\frac{1}{1+x^{2}}\\ \Phi =\sum \phi_{i} \end{array} \end{array}$$

(7) $$\begin{array}{l} \{\begin{array}{l} L=\sum_{x}\left(w_{lum}\left(x\right)\cdot I\left(x\right)\right)\\ w_{lum}=\frac{1}{1+x^{2}} \end{array} \end{array}$$

(8) $$\begin{array}{l} \{\begin{array}{l} w_{L}=\frac{\Phi_{L}L_{L}}{\Phi_{L}L_{L}+\Phi_{R}L_{R}}\\ w_{R}=\frac{\Phi_{R}L_{R}}{\Phi_{L}L_{L}+\Phi_{R}L_{R}} \end{array} \end{array}$$

### 3.2. Object Recognition and Localization on the CPM

The discharge of neurons in CPM reflects the luminance information from stereovision. The task requires the model to recognize an object's or end-effector's luminance patterns and return their locations within the CPM. The heuristic used here assumes that an object is usually observed by a closed contour, and the center of this contour might be used to represent the location of this object. Since the focus of this work is on sensorimotor coordination rather than complex object recognition, the more complex cases of occlusion and boundary ownership are not taken into account (Heydt et al., 2003; Layton and Yazdanbakhsh, 2015; Dresp-Langley and Grossberg, 2016). To achieve our goal, we first applied a center-surround filter and convolved CPM's luminance profile regardless of depth, as shown in the grid in Figure 2D, where the filled pattern is an example and corresponds to what we actually used in the experiments. This filter is able to reduce the noise and enhance the edges. After that, a Gaussian filter with size of (5,5) was used to suppress the noise in the map. The Gaussian filter is defined by


(9) $$\begin{array}{l} G_{i}=\frac{\exp\left(-{\left(d_{i}-2\right)}^{2}\right)/2.42}{\sum_{i=1}^{25}G_{i}} \end{array}$$

where *i* is the index of values on the filter kernel and can be from 1 to 25, and *d*_*i*_ is *ith* value's position on the kernel relative to the center and can be 1, 2, or 3. After this, a threshold filter eliminated the discharges of all neurons on the map whose discharges represented luminance values below 130 *cd*/*m*^2^. The resulting map was tested to be clean enough for object localization based on clustering approaches. Luminant spots emitted from an identified object are clustered with the same label through K-means algorithm, and the returned objects' cluster centers are used as their position in CPM.

### 3.3. Motor Planning: Neural Networks

**Figure 3:**
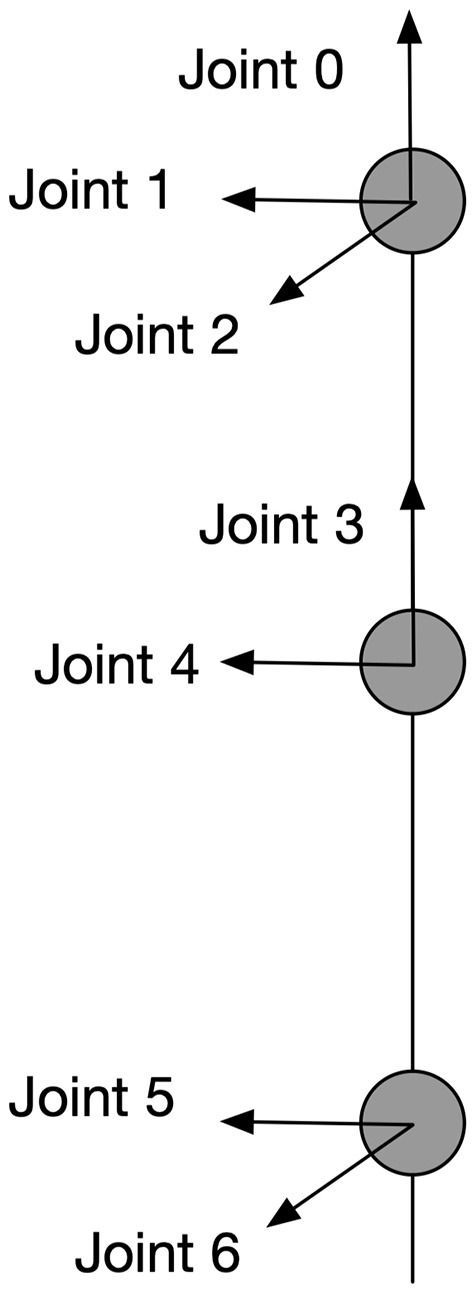
Kinematic information of the arm model. This is a 7-DOF arm with each joint and its rotation axe shown in the figure. All the angular rotations in this paper are in terms of counter-clockwise except for joint 2 which works clockwise.

Visual processing and recognition stages compute the spatial information of the target-object and the end-effector and encode this information as the position of discharging neurons in the cyclopean map. Our model connects the neurons in the cyclopean map via adaptive synaptic connections to motor neurons to guide the movement of a humanoid arm with 7 degrees of freedom (DOF) and let the end-effector (wrist) reach arbitrary positions that are reachable and visible. The arm model is shown in Figure 3. Interactions of two functionally complementary subsystems are needed to process this: one subsystem controls the upper limb (position controller) and the other subsystem adjusts postures in response to the environmental conditions (posture controller). This mechanistic property is in line with physiological findings showing that two main systems in human parieto-frontal networks play a major role in visually guided hand-object interaction (Lega et al., 2020). These two systems are associated with controlling upper-limb positions and with coding hand postures, respectively.

#### 3.3.1. Position Controller

The position controller works as a modified version of Grossberg-Bullock reaching model (Bullock et al., 1993), where direction-tuned neural networks code a 3-DOF arm to successfully reach spatial targets with a satisfactory error on a 2D working surface. Here we expand it with an additional dimension and make the modified model capable of taking spatial information from CPM and to send movement control signals to arm's joints 0, 1, 2, and 4 accordingly. Two neural networks are embedded: a position-tuned net (PTN) possessing neurons sensitive to specific spatial zones in CPM; and a supplementary direction-tuned net (DTN) possessing neurons sensitive to specific ranges of spatial direction vectors. In practice, PTN generates the first command every time when a new target appears, followed by DTN which corrects the arm's positions based on the spatial vectors from the end-effector to the target.

**Figure 4:**
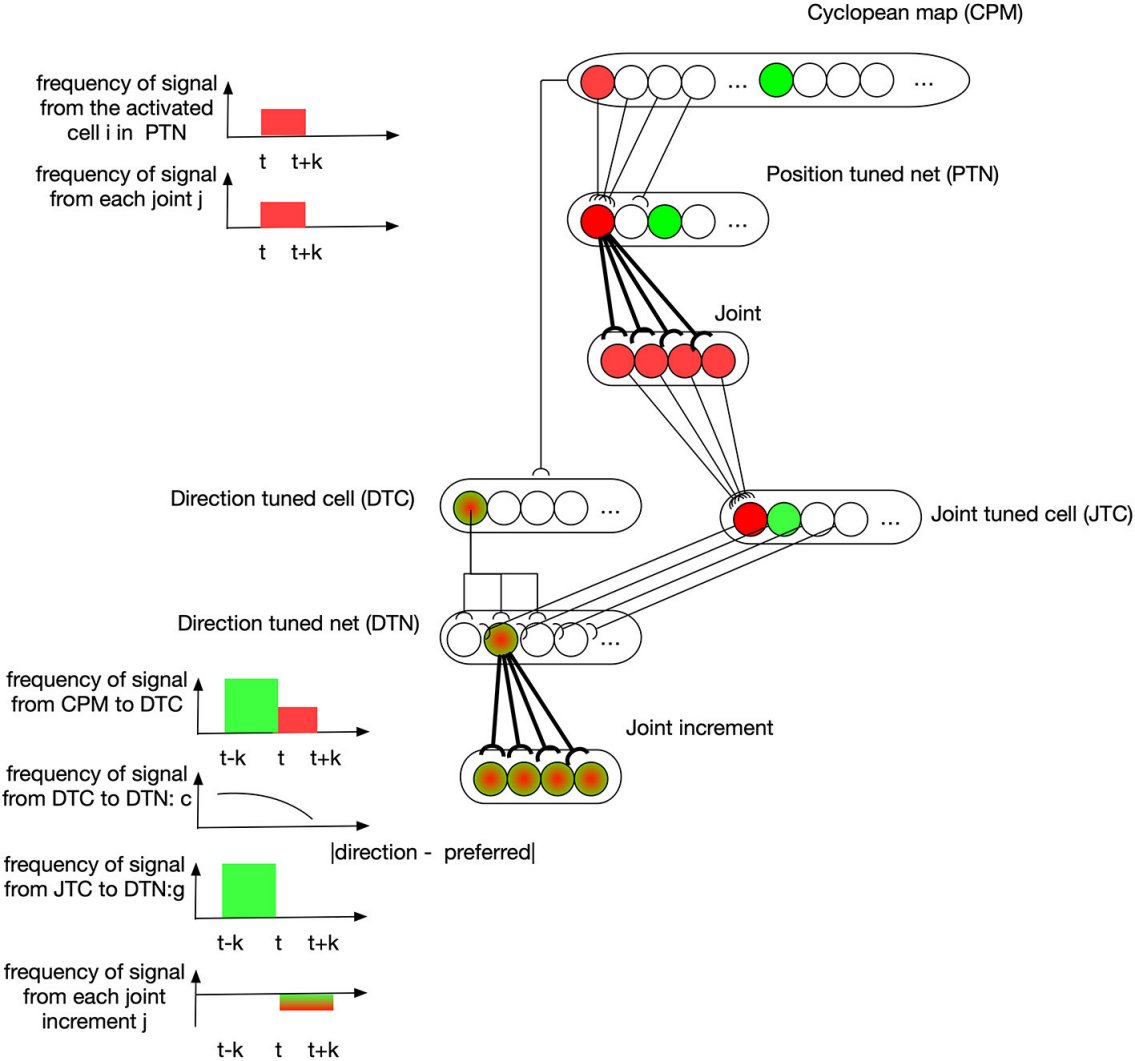
Position controller: learning of neural networks. At the time t-k, the visual information of the end-effector activates the green cell in the CPM. Then the end-effector is moved to another position represented by the red cell in CPM at time t. A cell in PTN down-samples the CPM by selectively receiving signals from a group of spatially-neighboring neurons in the CPM. At the same time, the state of arm joints stays in response to the red PTN cell, and this simultaneous activation of both PTN cell and joint cell strengthens the synapses between them through associative-learning rules (bolded connections between PTN and joint neurons in the figure). In the subsequent motion of the end-effector, the direction of motion is captured by some DTC cells, as shown by a green-red cell for example, because this direction lies in their receptive fields. Their discharges are dependent on the angular difference between their preferred direction and the perceived direction. A cell in DTN receives two signals, one from a DTC and the other from a JTC, leading to a selective tuning for a specific joint configuration. The number of cells in DTN equals the product of the number of cells in DTC and JTC so that DTN captures all possible situations. A DTN cell's discharge is equal to the multiplication of two signals it receives. At time t, DTN cells receive JTC's signals in green state and the DTC signals capturing the end-effector's motion direction. The synapses between activated DTN cells and the joint increments resulting in this motion are then learned accordingly.

An example describing how learning takes place in these two neural nets is depicted in Figure 4. At the time t-k, the visual information of the end-effector activates the green cell in the CPM. Then the end-effector is moved to another position represented by the red cell in CPM at time t. A cell in PTN down-samples the CPM by selectively receiving signals from a group of spatially-neighboring neurons in the CPM. At the same time, the state of arm joints stays in response to the red PTN cell, and this simultaneous activation of both PTN cell and joint cell strengthens the synapses between them through associative-learning rules (bolded connections between PTN and joint neurons in the figure). In the subsequent motion of the end-effector, the direction of motion is captured by some DTC cells, as shown by a green-red cell for example, because this direction lies in their receptive fields. Their discharges are dependent on the angular difference between their preferred direction and the perceived direction. A cell in DTN receives two signals, one from a DTC and the other from a JTC, leading to a selective tuning for a specific joint configuration. The number of cells in DTN equals the product of the number of cells in DTC and JTC so that DTN captures all possible situations. A DTN cell's discharge is equal to the multiplication of two signals it receives. At time t, DTN cells receive JTC's signals in green state and the DTC signals capturing the end-effector's motion direction. The synapses between activated DTN cells and the joint increments resulting in this motion are then learned accordingly.

Mathematically, using *PTN*_*ij*_ to represent the synaptic weight from a PTN cell *i* to a joint *j* (*j* = 0, 1, 2, 4), the learning rule for PTN is described by Equations (10) and (11). With a desired position pos^*^, PTN generates a motor command by Equation (12), where ${\hat{p}}_{i}$ is cell *i* 's sensitive position zone in BCM, *epos* is the end-effector's position in BCM, θ_*j*_ is joint j's position in degrees. η was 0.5 in our simulation.


(10) $$\begin{array}{l} p_{i}\left(t\right)=\{\begin{array}{ll} 1, & if\;epos\left(t\right)\;in\;{\hat{p}}_{i}\\ 0, & otherwise \end{array} \end{array}$$

(11) $$\begin{array}{l} PTN_{ij}\left(t+1\right)=PTN_{ij}\left(t\right)+p_{i}\left(t\right)\cdot \left(\left(1-\eta \right)\cdot \theta_{j}\left(t\right)+\left(\eta -1\right)\cdot PTN_{ij}\left(t\right)\right) \end{array}$$

(12) $$\begin{array}{l} \theta_{j}\left(0\right)=PTN_{ij},i\;:\;pos^{*}\;in\;{\hat{p}}_{i} \end{array}$$

Using *DTN*_*i,j*_ to represent the synaptic weight between a DTN cell *i* to a joint *j* (*j* = 0, 1, 2, 4), the learning rule for DTN is given by Equations (13)–(17), where *adif*(*v*_1_, *v*_2_) is the angular difference between two vectors *v*_1_ and *v*_2_, ${\hat{v}}_{i}$ represents the spatial direction range for which cell *i* is tuned, ${\hat{\theta }}_{ij}$ is cell *i*'s sensitive angular range of joint *j*, *t* is time or step in the learning and testing dynamics, *c*(v, v^*^) is a direction-tuned neuron's tuning curve given a stimulated vector *v* and its selective vector v^*^, and *a* → *b* represents a spatial vector defined by position *a* and *b* with a direction from former to latter.

After enough learning, DTN is expected to have the ability to drive the arm to move the end-effector to a desired position pos^*^ by Equation (18). In the above functions, both γ and ρ are step-sizes and δ is a parameter of regularization. β represents the tuning activity of DTN neurons. In our simulation, we used γ = 1, δ = 0.1, ρ = 0.05, and β = 0.001.


(13) $$\begin{array}{l} dv\left(t\right)=epos\left(t\right)\to epos\left(t+1\right) \end{array}$$

(14) $$\begin{array}{l} △\theta_{j}\left(t\right)=\theta_{j}\left(t+1\right)-\theta_{j}\left(t\right) \end{array}$$

(15) $$\begin{array}{l} c\left(v,v^{*}\right)=\{\begin{array}{ll} \exp\left(-\beta \cdot adif{\left(v,v^{*}\right)}^{2}\right), & if\;adif\left(v,v^{*}\right)<90{}^{\circ}\\ 0, & otherwise \end{array} \end{array}$$

(16) $$\begin{array}{l} g_{i}\left(t\right)=\{\begin{array}{ll} 1, & if\;\theta_{j}\left(t\right)\;in\;{\hat{\theta }}_{ij}\;for\;j=0,1,2,4\\ 0, & otherwise \end{array} \end{array}$$

(17) $$\begin{array}{r} DTN_{ij}\left(t+1\right)=DTN_{ij}\left(t\right)+\gamma \cdot c\left({\hat{v}}_{i},dv\left(t\right)\right)\cdot g_{i}\left(t\right)\cdot (△\theta_{j}\left(t\right)\\ -\delta \cdot DTN_{ij}\left(t\right))) \end{array}$$

(18) $$\begin{array}{l} \theta_{j}\left(t+1\right)=\theta_{j}\left(t\right)+\rho \cdot DTN_{ij},i\;:\;\{\begin{array}{l} \theta_{j}\left(t\right)\;in\;{\hat{\theta }}_{ij}\;for\;j=0,1,2,4\\ \underset{i}{\min}\;adif\left({\hat{v}}_{i},epos\left(t\right)\to pos^{*}\right) \end{array} \end{array}$$

#### 3.3.2. Posture Controller

**Figure 5:**
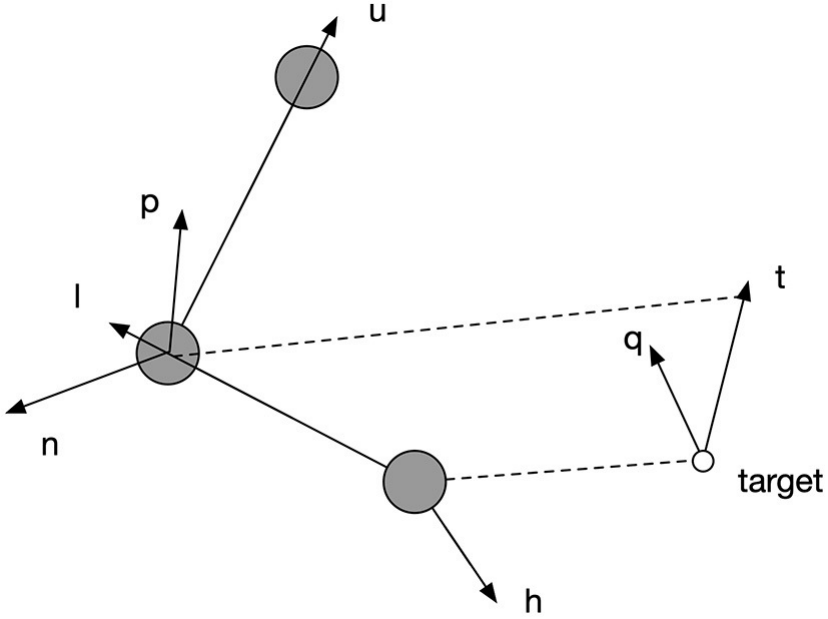
The explanation of spatial relations by which the posture controller works. Vectors $\vec{u}$, $\vec{l}$, $\vec{h}$ are along the spatial directions of upper arm, lower arm and hand, respectively. Vectors $\vec{n}$ and $\vec{p}$ are the normal vectors of planes defined by $\left(\vec{u},\vec{l}\right)$ and $\left(\vec{l},\vec{n}\right)$, respectively. Vector $\vec{t}$ represents the desired direction when the end-effector reaches the target. $\vec{q}$ is the normal vector of the plane defined by $\left(\vec{l},\vec{t}\right)$.

Figure 5 explains the functioning of the posture controller, which adjusts the orientation of the palm to parsimoniously reconcile the environmental requirement defined by specified geometrical relations. Vectors $\vec{u}$, $\vec{l}$, $\vec{h}$ are along the spatial directions of upper arm, lower arm and hand, respectively. Vectors $\vec{n}$ and $\vec{p}$ are the normal vectors of planes defined by $\left(\vec{u},\vec{l}\right)$ and $\left(\vec{l},\vec{n}\right)$, respectively. Vector $\vec{t}$ represents the desired direction when the end-effector reaches the target. $\vec{q}$ is the normal vector of the plane defined by $\left(\vec{l},\vec{t}\right)$. The rotation of each joint is defined by the angular difference of a pair of vectors as shown in Equation (20). The rotation of joint 3 in degrees was defined to be equal to the angular difference between $\vec{p}$ and $\vec{q}$, so that when end-effector reached the target, the plane of the palm could be perpendicular to the plane formalized by lower arm and target direction. After this, joint 6 can be rotated to align the palm to the target direction by a degree equal to the angular difference between $\vec{l}$ and $\vec{t}$. However, due to limited rotation capacity of all the joints, the hand might not perfectly align with the target's direction; so joint 5 can further adjust the hand's direction by rotating by a degree equal to the angular difference between $\vec{h}$ and $\vec{t}$ at last to make the alignment as close as possible. However, each joint is limited in its range of rotations. If the desired rotation angle exceeds their limit, they rotate up to the maximum or minimum of the range, as shown by Equations (19) and (20).


(19) $$\begin{array}{l} \{\begin{array}{l} \vec{n}=\vec{u}\times \vec{l}\\ \vec{p}=\vec{l}\times \vec{n}\\ \vec{q}=\vec{t}\times \vec{l} \end{array} \end{array}$$

(20) $$\begin{array}{l} \{\begin{array}{l} joint\;3=adif\left(\vec{p},\vec{q}\right),joint\;3\in \left[0,105{}^{\circ}\right]\\ joint\;5=adif\left(\vec{h},\vec{t}\right),joint\;5\in \left[-15{}^{\circ},15{}^{\circ}\right]\\ joint\;6=adif\left(\vec{l},\vec{t}\right),joint\;6\in \left[-50{}^{\circ},50{}^{\circ}\right] \end{array} \end{array}$$

## 4. Biological Evidence

The learning in this model is autonomous, unsupervised, and local. The model autonomously generates movements, by which the activities in sensory and motor representations can be associated to predict each other. This learning procedure is inspired by multiple studies suggesting infants acquire spatial and motor knowledge and their associations by self-exploration and object manipulation (Needham, 2000; Soska et al., 2010; Schwarzer et al., 2013; Soska and Adolph, 2014). Like infants, the model learns autonomously in an unsupervised manner. The learning equations are not based on error-correction following teacher-provided learning targets but rather on associative learning, which simply associates correlated sensory and motor activities. Learning feedback is provided directly by the environment through action-perception loops. This type of sensorimotor organization is believed to underly the more abstract concepts of space. For instance, some investigators found that, after object exploration, infants' performance in mental spatial imagination is improved, which suggests an importance contribution from exploration experience to spatial development (Slone et al., 2018). The sensory representation herein is projected in CPM created by two retinal luminance maps. This stereoscopic sensing recovers the depth information through a specified geometrical definition based on binocular disparity and enables the motion detection in three-dimensional aspects. This three-dimensional motion sensing has been investigated using various of paradigms including direction selectivity, temporal resolution and changing size, etc. (Beverley and Regan, 1974; Gray and Regan, 1996; Portfors and Regan, 1997). Among those studies, Beverley recorded electrical brain responses to stimuli in motion along the depth-axis and found these responses to be different with respect to different binocular disparities. The explicit map representations in the model allow local learning. Maps provide a representation for space and sensitivity-zones (e.g., Equation 10) determine local regions in this space. For example, learning for PTN cells occur only when the end-effector is within their local sensitivity zone (Equations 10 and 11). That way highly nonlinear relationships across the entire space can be simplified by local approximations, resulting in a much simpler learning approach.

One important technique used in this model is to coordinate spots on two retinas by spatial-frequency similarity. The spatial-frequency channels in human vision and their psychometric functions are well known (Sachs et al., 1971). On the motor-control side, our model has neurons selectively tuned to different spatial directions, abstracting neurons identified in the primary motor cortex (M1). In one study that reported recordings from monkey's motor cortex, researchers found cells that code the direction of movement in a way dependent on the position of the arm in space (Caminiti et al., 1990). Similarly, DTN in our model combine information from both arm configuration and motion direction. More recent studies reported similar neurons found in human cortex M1 (Tanaka et al., 2018; Feldman, 2019).

## 5. Experiments

### 5.1. Platform and Simulation Procedures

**Figure 6:**
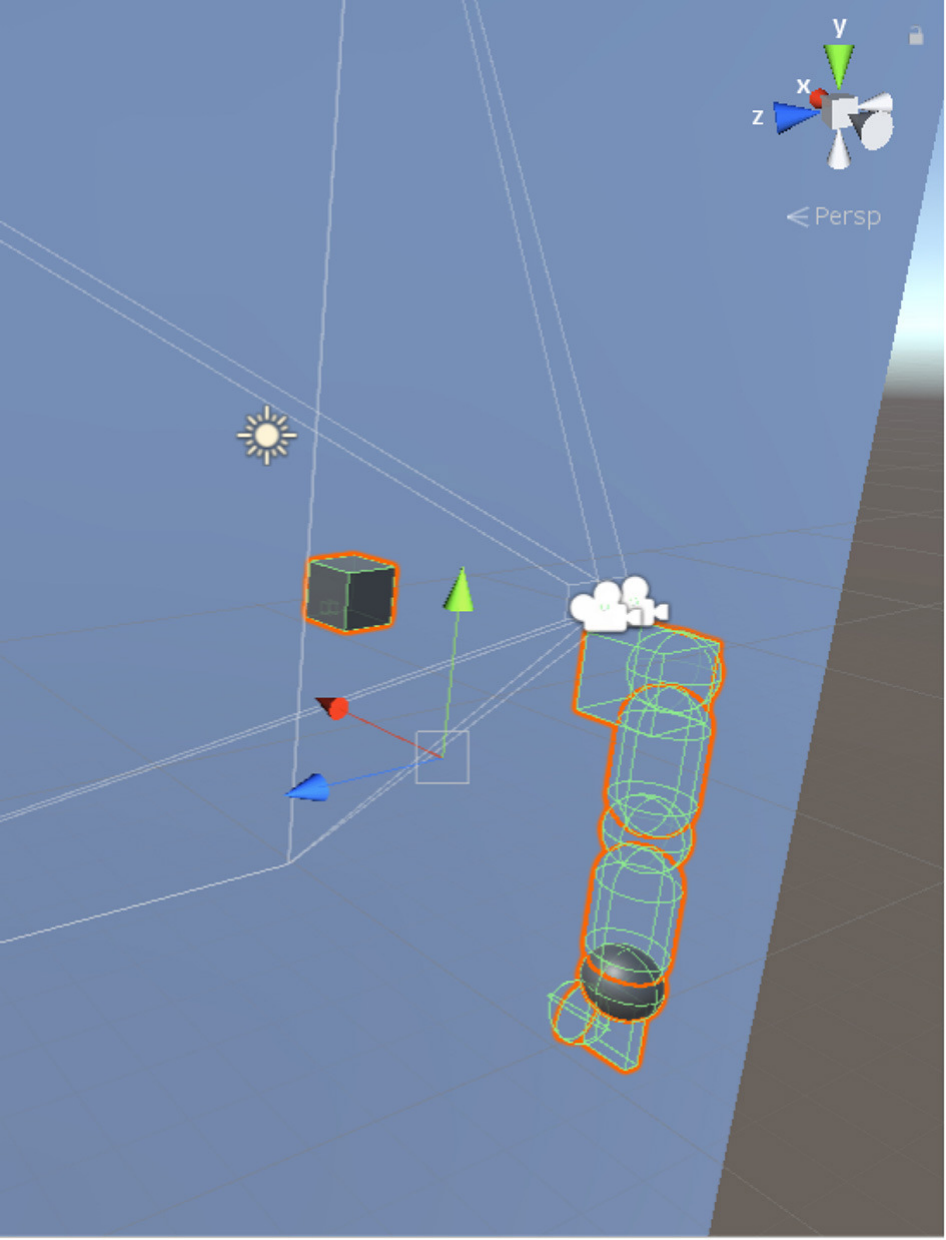
Simulation environment in Unity3D. The axes indicated by the green and the red arrows are aligning with the axes marked by joint 2 and joint 0 in Figure 3, respectively. The blue arrow is pointing inward the shoulder while joint 1 in Figure 3 is pointing outward the shoulder.

Our model was simulated on Unity3D, where we programmed the objects in the environment to make the arm work following the kinematic rules described in this paper. We calibrated the measurements by assuming that a unit scale in Unity3D scene is equal to 10 cm. As shown in Figure 6, a pair of cameras were placed both in 10 cm upward than the center of shoulder joint and 9.5 cm and 14.5 cm leftward than the center of shoulder joint, respectively. These cameras took pictures at a resolution of 300*300 pixels. The gaze position of two eyes was 120 cm forward, 15.7 cm rightward, and 13.7 cm below the left eye. We used capsules with a diameter of 10 cm for upper and lower limbs and spheres with equal diameter as the joints connecting two equally long arm limbs. The end-effector herein was the wrist that is the only visible body part. We kept other limbs transparent due to the requirement of precise prediction of end-effector's spatial position during visual processing. We also placed a cube with 10 cm long, 5 cm wide and 2 cm thick to serve as the palm. The lengths of limbs were 28 and 10 cm, respectively.

We trained the model using a uniformly distributed random-variable that generated self-exploratory movements. The neural networks embedded in the model learned spontaneously in the way described. The model was tested by using a black cube as target with sides 10 cm long placed randomly somewhere within the view range of “eyeballs” at the beginning of each trial. The system was then expected to deliver the end-effector to contact the cube, and when it reached the cube, the palm was expected to point in the forward direction as the red arrow axis shown in Figure 6.

### 5.2. Validation of Visual Processing

**Figure 7:**
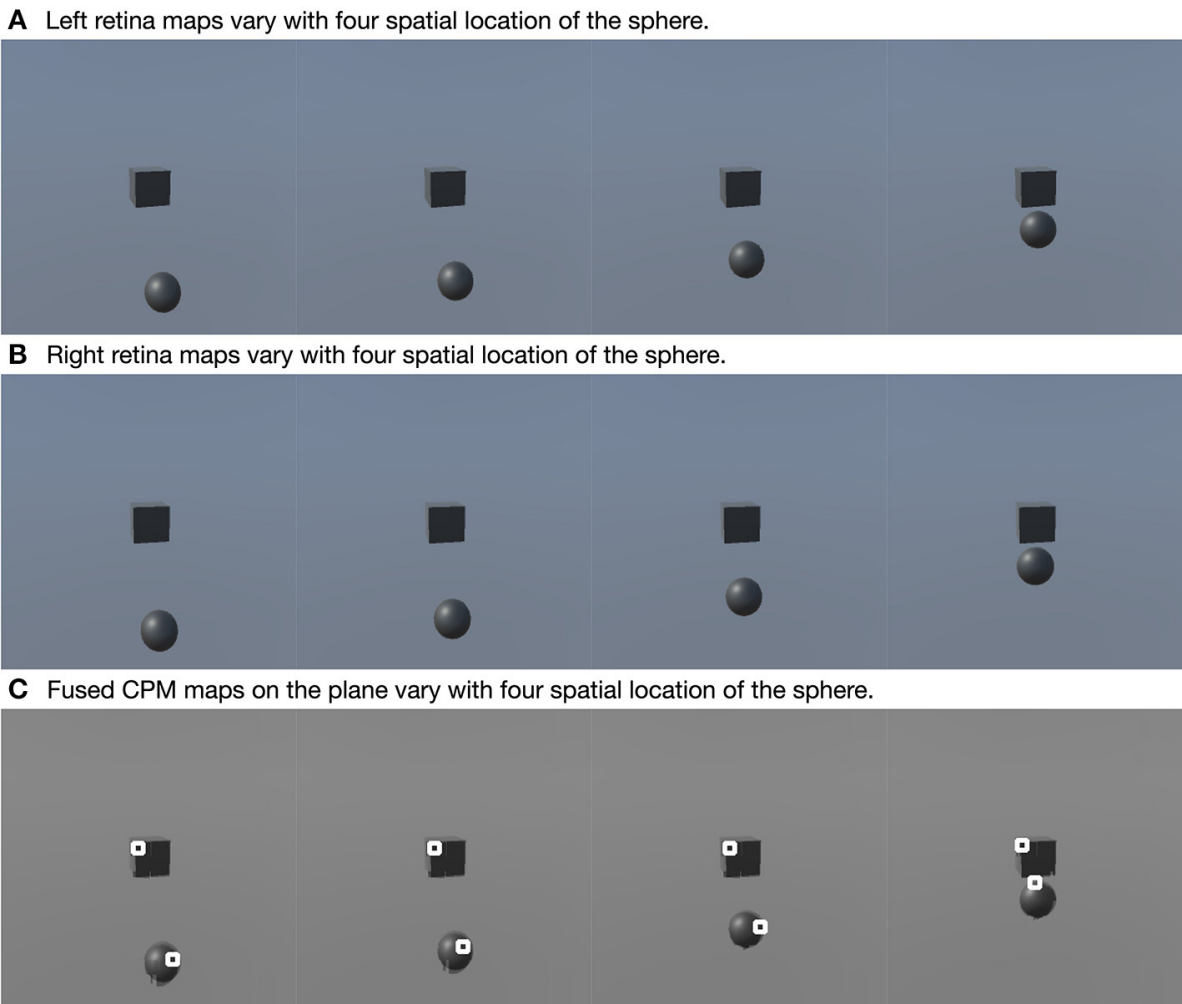
Examples of visual localization. This figure contains three rows and four columns. Each column indicates an example, and three rows are left retina map **(A)**, right retina map and fused CPM's projection on the plane **(B,C)**. In the third row, two white circles are marked on two objects, respectively. These marks indicate the spatial location of two objects determined by the model.

**Figure 8:**
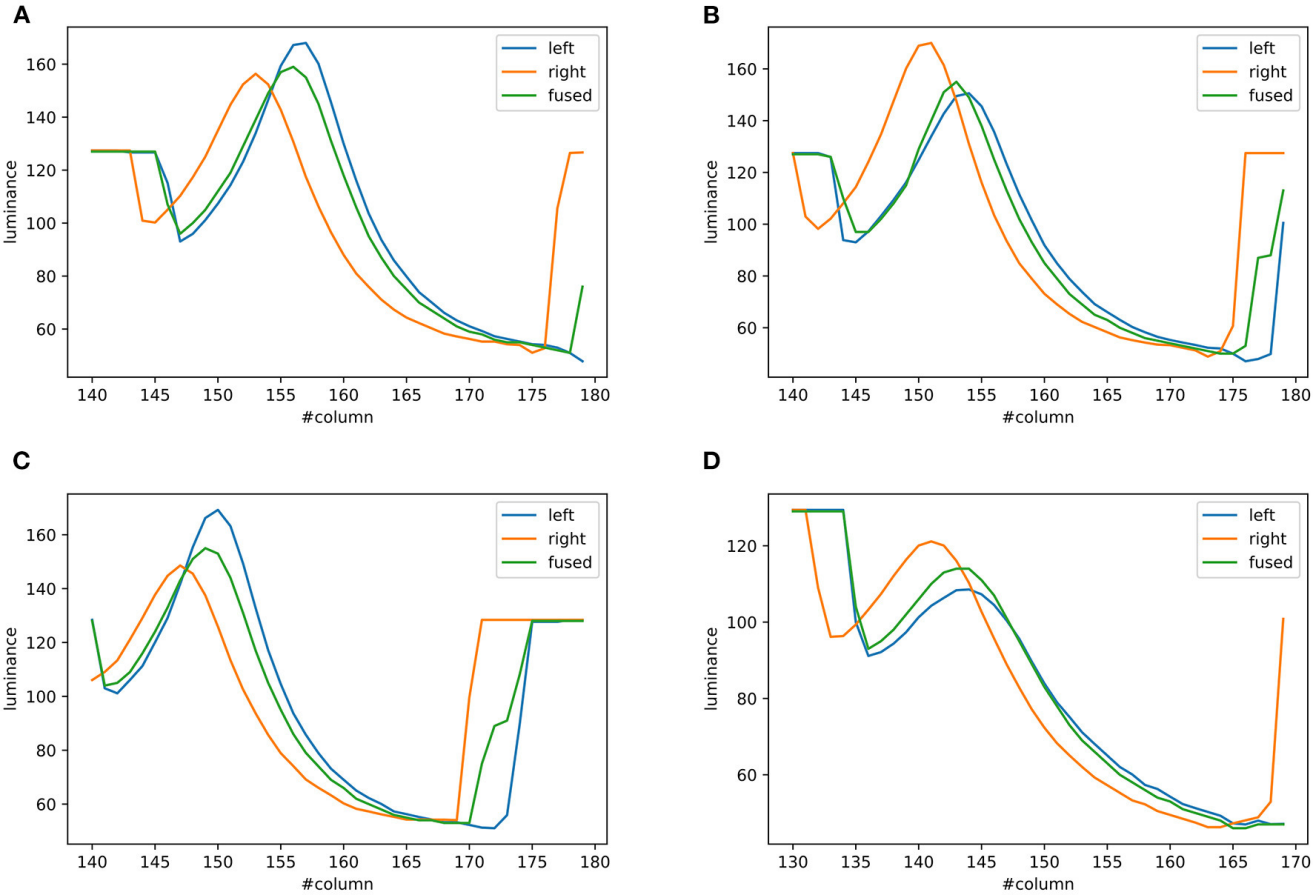
Examples of binocular combination. The four **(A–D)** show the luminance profiles of a single row where the four black spheres in Figure 7 are located, respectively. Each figure shows the luminance profiles from the left retina, the right retina and the fused map.

**Figure 9:**
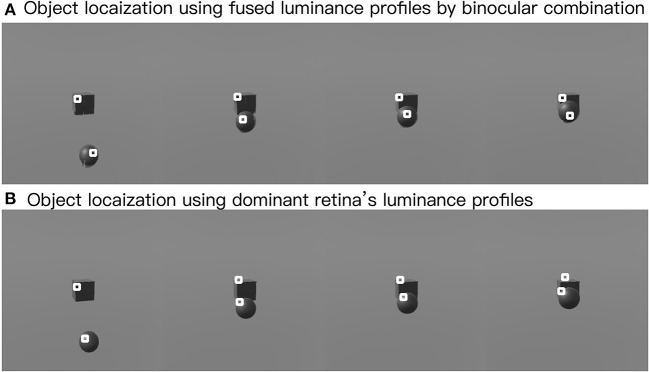
Effects of clustering in binocular combination. The upper row shows the clustering after binocular combination **(A)**, and the lower row shows the clustering using the dominant eye's luminance profile **(B)**. White circles mark the cluster centers.

**Figure 10:**
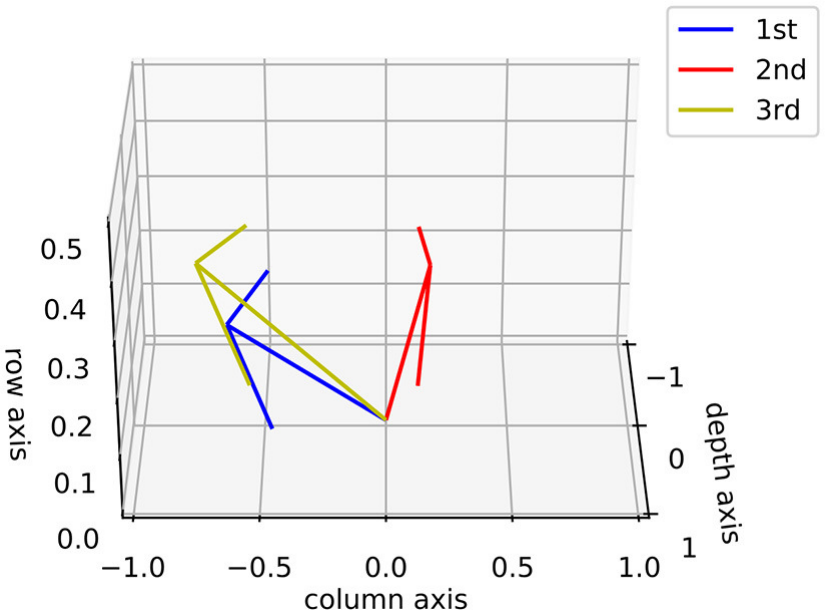
Examples of three-dimensional motion direction. This figure shows three spatial vectors along the column axis, row axis and depth axis, correspondent to the three motion represented in Figure 7.

In this part, we show the feasibility of the visual processing methods by an example containing three movements. As shown in Figure 7, two cameras representing two eyeballs were fixed to the center of the black cube. The cameras were kept stable, while the black sphere was moving for three steps. Eight pictures from four states and two retinas were captured. First, we picked a row of the pixels and plotted its luminance with respect to the index of columns for each of the pictures in Figure 7. As shown in Figure 8, binocular disparities co-varied with the four motion states of the black sphere, as indicated by variations in the misalignments between luminance curves from left and right retinas. As indicated by Equations (1, 2), this model is dominated by the left eye as the luminance profiles are compensated from the right eye to the left eye. The fused profiles' shape is in line with the dominant eye in terms of spatial locations, while the contrast change and absolute luminance value are both rebalanced taking both eyes into account. Second, the object localization presented with the white circles in the bottom row of Figure 7 reflects their real spatial information. It can also be found that, with illumination unchanged, objects' recognized centers are locally stable. Even when two objects are close to each other, the model is able to differentiate them. Third, results also show examples about the effect of contrast-related weighted summation of binocular luminance profiles. In Figure 9, we compared binocular combination with dominant vision in the results of localization. As discussed, Ding-Sperling's model of contrast rebalance optimizes contrast-based localization. Specifically, this method localizes the object on the edges and corners, or on the zones of chiaroscuro of smooth surface. This also stabilizes the localization of objects in motion, as well as when two objects are spatially close. In contrast, monocular vision is more sensitive to the absolute luminance value as objects tend to be localized on the light spot. When two objects are close to each other, the cluster center of the black cube is marginalized, as shown in Figure 9. Last, Figure 10 shows the three-dimensional spatial vectors indicating these three motion directions in Figure 7. Putting the maps in Figure 7 into the 3D coordinates shown in Figure 10, the depth value of the cube is smaller than that of the sphere. The directions along the depth axis are reconstructed, which coincide with the real motion as the black sphere are moving toward the black cube.

### 5.3. Experiment 1: Reaching With Fixed Gaze Position

In this experiment, we fixed the gaze position as where it was initialized. We tested the functions of each module as well as the neural networks. In this case, the objects and the arm were all lying on the peripheral retina during the test session. We reported the performance in terms of the cartesian error between the wrist and the target, and the angular difference of the hand posture and the target orientation.

#### 5.3.1. Experimental Parameters and Learning

**Figure 11:**
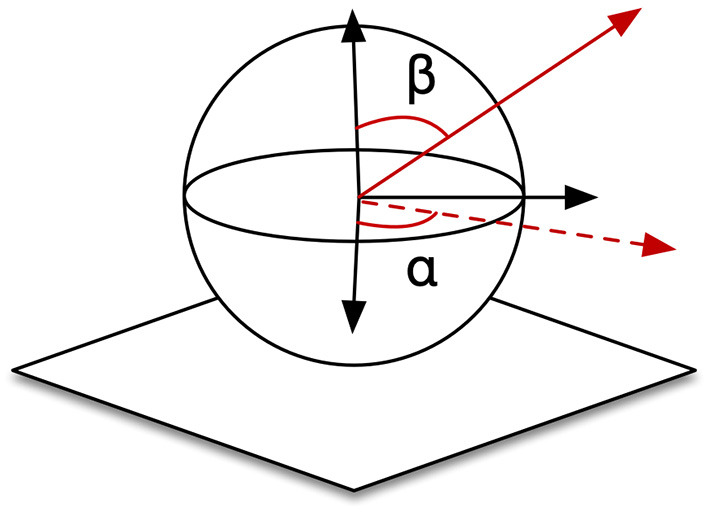
Definition of spatial vectors.

This system possesses two trainable neural-networks, TPN and DTN, where neurons are sensitive to positional zones in CPM, angular zones in arm joints or spatial directions in CPM. To implement in simulation, we demarcated the CPM of size (300,300,300) into 2.7 × 10^4^ cube zones of size (10,10,10) as the$\;{\hat{p}}_{i}$ in Equations (18)–(20). The ranges of joints driving the end-effector were [0°, 90°], [60°, 120°], [0°, 25°], and [0°, 90°] for joints 0,1, 2, and 4, respectively. We used 6° as the interval to demarcate this hyperspace into 9,000 (15 × 10 × 4 × 15) angular zones, ${\hat{\theta }}_{i}$. Spatial vectors are represented using polar coordinates (α_*i*_, β_*i*_) here as shown in Figure 11. We defined 180 selective vectors, ${\hat{v}}_{i}$ in (18), by $\alpha_{i}=20{}^{\circ},\;40{}^{\circ},\;60{}^{\circ},\ldots ,340{}^{\circ},\;360{}^{\circ}$ and $\beta_{i}=20{}^{\circ},\;40{}^{\circ},\;60{}^{\circ},\ldots ,160{}^{\circ},\;180{}^{\circ}$. Therefore, DTN contains 1.62 × 10^6^(9, 000 × 180) cells because of its sensitivity to both spatial direction and arm position. PTN contains 2.7 × 10^4^ cells each selectively becoming active for a unique zone in CPM. In the learning session, a total amount of 2.916 × 10^7^ steps of self-exploration were implemented to drive the learning and self-organization processes of the two neural networks. We additionally tested the performance when the system was trained with 1/3 and 2/3 of the total amount. We also trained the system with an additional noisy condition, where a random noise in the range of 0 to 5 degrees was added to each of the joints.

#### 5.3.2. Tests and Results

In the testing session, the system took six groups of tests, within which each group contained 50 trials. Groups of tests are distinguished by three different amounts of learning and two conditions (with or without noise) to test robustness. In each trial, the system operated in the way described above and predicted the increments of arm joints to move the end-effector to a target-object placed in an pseudo-randomly assigned position that is reachable, visible, and also novel, i.e., not experienced during the learning history. The system was also expected to adjust the direction of the palm toward the forward directions. Importantly, in each trial the system was only allowed to move for four steps.

We evaluated the performance in terms of Cartesian error measured by the Cartesian-distance between the end-effector and the target object, as well as by the angular difference between the palm and the desired direction.

**Figure 12:**
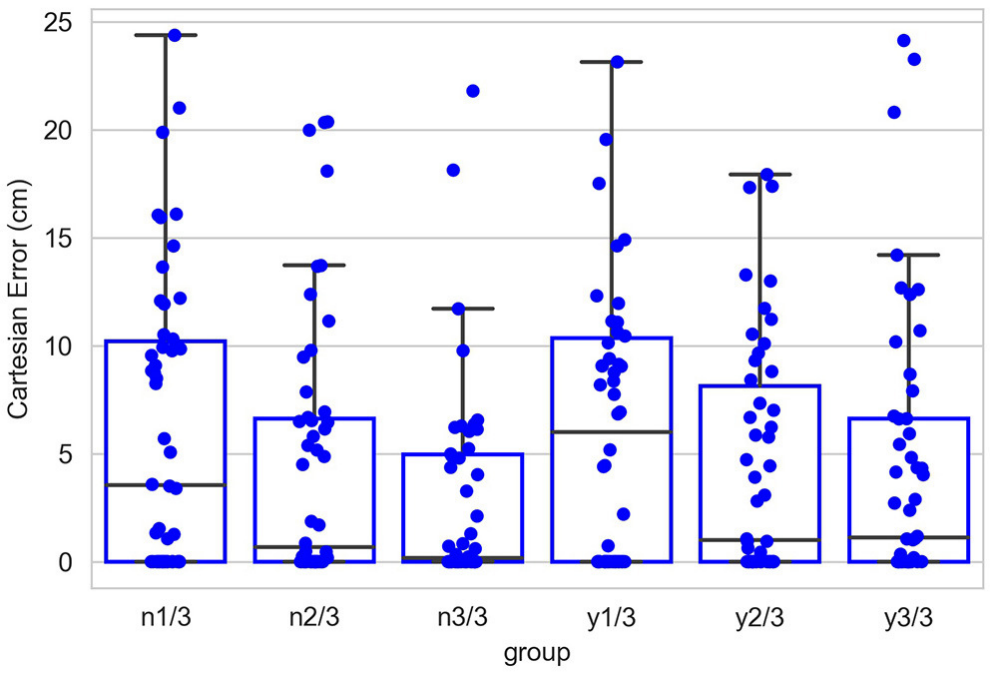
Cartesian errors in all six conditions. Along the vertical axis, “n” means results are obtained under the condition without noise applied to joints, “y” means noisy, and the fractions after n or y show the proportion of the entire learning session experienced after which test results are obtained. Within the chart, each box contains three black bars and a box body marks two levels by upper and lower edges. From up to down, these five levels indicate the maximum, third quarter (Q3), median, first quarter (Q1) and minimum values of the value set represented, which is called “five-number summary.” Points that past Q3+1.5*IQR (interquartile range) or Q1-1.5*IQR are not included in the box.

**Figure 13:**
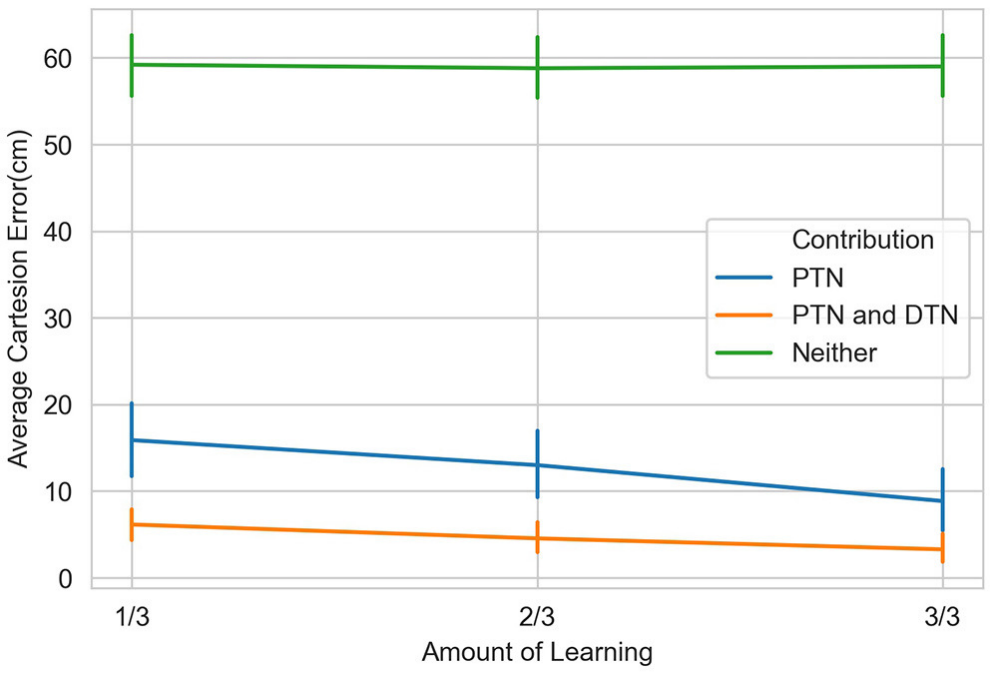
Contributions of position-tuned and direction-tuned neural networks. The three values indicated by the green line are 59.20, 58.80, and 59.00 cm, respectively from 1 to 3 of the learning session to the end of the full session. With PTN involved, as shown by the blue line, these three values are 15.89, 13.00, and 8.86 cm. When both PTN and DTN are functioned, these three values are reduced to 6.74, 4.55, and 3.28 cm.

Figure 12 shows the contribution of the position controller by experimental conditions. In this figure, along the vertical axis, “n” means results are obtained under the condition without noise applied to joints, “y” means noisy, and the fractions after n or y show the proportion of the entire learning session experienced after which test results are obtained. Within the chart, each box contains three black bars and a box body marks two levels by upper and lower edges. From up to down, these five levels indicate the maximum, third quarter, median, first quarter and minimum values of the value set represented, which is called “five-number summary.” Regardless the presence of noise, Cartesian error decreases with larger amounts of learning. The one-way ANOVA also shows the significant effect of the amount of learning (*F*(2, 297) = 4.98, *p* < 0.01). Comparing the conditions with and without noise, as expected, the median levels of Cartesian errors are higher when noise is present. The median error is 0.96 cm without noise applied and 2.54 cm with noise present, although we found no significant effect of the noise condition on the Cartesian error [*F*_(1,298)_ = 1.49, *p* = 0.22]. Moreover, the first quarter error values are 0 for all six conditions, and median errors are smaller than 5 cm for all conditions except for the noisy one after 1/3 of the learning session (6.01 cm). In particular, after going through the entire learning session, under the condition without noise, the median Cartesian error is 0.16 cm, and the third quarter error value is 4.96 cm. These results indicate that the position controller is able to deliver the end-effector to contact or reach the target with satisfactory error levels, compared to other recent studies (Mahoor et al., 2016; Nguyen et al., 2019; Rayyes et al., 2020). As an example, Mahoor reported a median Euclidean distance error of approximately 4 cm achieved by neural-networks learned through motor babbling. Note that the accuracy and precision of the system can be improved significantly by increasing the resolution of internal representations and the learning trials (circular reactions). Here we demonstrated that even with low resolution and fast learning, the network is capable of reasonable performance levels. Figure 13 further shows the contributions of position-tuned net and direction-tuned net, respectively, considering all conditions. After full learning experience, PTN alone reduces the average distance error from 59 to 8.86 cm, and followed by DTN who finally reduces this average error to 3.28 cm. Comparing PTN only and PTN&DTN, there is a significant effect of DTN [*F*_(1,298)_ = 37.15, *p* < 0.01]. This suggests that the PTN successfully drives the end-effector to somewhere close to the target, and DTN also behaves effectively in correcting end-effector's position.

**Figure 14:**
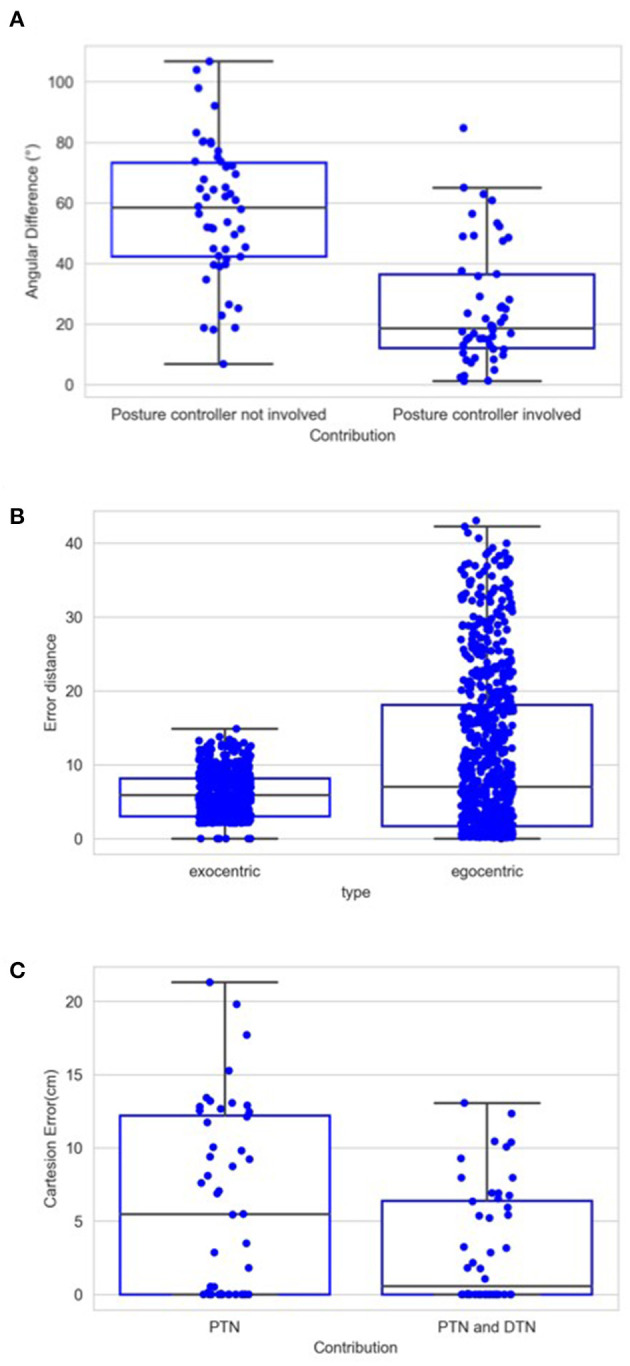
**(A)** Average angular errors between palm and desired direction when trials are completed with and without the application of posture controller. Same as Figure 12, each box in this chart summarizes five numbers. For the left box, these numbers are 106.69, 73.30, 58.36, 42.30, and 6.80 in degree from maximum to minimum, respectively, and 84.73, 36.31, 18.63, 12.06, and 1.16 for the right box. **(B)** Distance error in terms of the number of cells in the CPM. For the left box, these numbers are 43.04, 18.11, 7.04, 1.69, and 0 from maximum to minimum, respectively, and 14.86, 8.14, 5.87, 3.04, and 0 for the right box. **(C)** Cartesian errors when PTN worked only and when both PTN and DTN worked. Same as Figure 12, each box in this chart summarizes five numbers. For the left box, these numbers are 21.29, 12.2, 5.46, 0, and 0 in cm from maximum to minimum, respectively, and 13.06, 6.39, 0.55, 0, and 0 for the right box.

Figure 14A shows the contribution of posture controller under the condition of full learning and without noise. When this component doesn't function, which means palm's direction keeps aligning with lower limb's direction, the median value of angular errors is 58.36°. This median error is reduced to 18.63° with the involvement of posture controller. The effect of posture controller is also significant [*F*_(1,98)_ = 55.76, *p* < 0.01]. This suggests that the posture controller is effective in adjusting palm's posture. In some positions, the desired directions might be awkward for the posture controller to adjust when those can possibly exceed joints' rotation limits.

These results in visually guided reaching demonstrate the effectiveness of sensorimotor coordinations and the three-dimensional exocentric external frame of references.

### 5.4. Experiment 2: Reaching With Active Fixation

**Figure 15:**
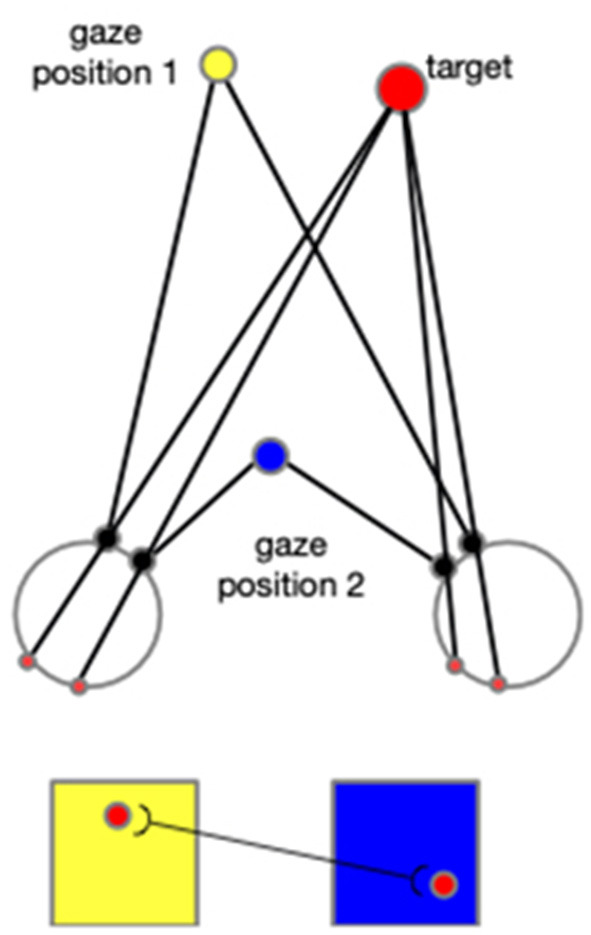
When the eyeballs rotate to fix on the gaze position 2 from the previous fixation (gaze position 1), a spot's projection on the two retinas moves accordingly. Two cells activated sequentially from the corresponding two CPM are then associated. In this figure, the yellow square represents the CPM of gaze position 1, and the blue square represents the CPM of the gaze position 2. The red disks represent the target and its projections on retinas and CPMs.

In addition to the first experiment, in which only the arm consists of degrees of freedom, we conducted a supplementary experiment adding the motion of eyeballs. By this experiment, we show how the neural networks are expanded when other body joints are included, such as eye movement, head rotation, and other body motions. As explained in the following context, adding any other degree of freedom would necessitate expansions in the same way as in the introduction of eye movement to the model. The cells in each neural layer would not only tune to the arm configurations and the directions, but they would also tune to the gaze positions. This means there would be different layers of cells, and each layer will correspond to a specific gaze position. When the reaching begins, the gaze position firstly triggers the selected layer, and then the cells within this layer then function according to the input arm configurations and directions. In addition, the CPM formalized by each gaze position will compensate each other in a way that cells from different CPMs that are projected by the identical spot in the environment are connected. As shown in Figure 15, this associated learning makes the spatial localization independent of the gaze position, and thus exocentric.

Regarding the eyeballs' rotation, there are two strategies to cope with the reaching behavior: (1) reaching a target without fixing on it; and (2) fixing on the target and then reach it. This experiment will be tested using the latter, since the former are shown in the first experiment.

#### 5.4.1. Illustration of the Eyeball Rotation Compensation

To illustrate the operation of exocentric representations, we conducted a separated test, in which we compared the errors of spatial localization under both exocentric and egocentric conditions. We tested internal representations of spatial position with two gaze positions and 740 spatial positions. The eyes rotated either 6° or 12° horizontally, with left eye rotating rightward and right eye rotating leftward. We used 3° as the resolution of the CPM cells considering the gaze positions. We compared the distance between target spots' internal representation before and after the rotation with and without the compensation. The results are shown in Figure 14B. The mean error in the egocentric condition is 11.06, which is reduced to 6.01 by the compensation. Due to the limited resolution regarding the gaze position and the size of the CPM, eyeball rotation have very little impact on some spots' projections, which can be found in the minimum error in the egocentric condition. However, the maximum and third quarter values of the error are greatly reduced. This means that, for the scenarios where eye movements cause drastic spatial localization errors, the compensation effectively reduces the errors in the localization. The ANOVA also shows a significant difference between these two groups of the errors [*F*_(1,1478)_ = 146.83, *p* < 0.01].

#### 5.4.2. Experimental Parameters and Learning

This experiment used the same parameters relevant to the CPM and the cell's tuning properties as what we used in the first experiment. However, since the eye movement was included, both PTN and DTN were expanded by adding cells with gaze position tuned properties, which selectively respond to a specific eyeball configuration represented by the vertical rotation, left eyeball horizontal rotation, and right eyeball horizontal rotation. We considered 0° for an eyeball's both horizontal and vertical rotations when the eyeball points exactly forward. The range of these rotations that the cells were tuned to were [−40°, 0°], [0°, 30°], and [−20°, 20°], respectively, with an interval of 10°. (Here we use positive values for upward and rightward rotations, and negative for downward and leftward rotations.) The cells thus were tuned to 100 (5 × 4 × 5) gaze positions, making the cells arranged in 100 different layers. Within each layer, the cells selectively discharge to 4,096 (8^4^) arm configurations. These are given by 8 angles for each of the four joints: 0°, 25°, 50°, …, 150°, 175°. Therefore, within each of the 100 layers, DTN contains 730, 800 (4, 060 × 180) cells and PTN contains 2.7 × 10^6^ cells. Importantly, from the first experiment to this one, we changed the resolution of arm configuration in each joint from 6° to 25°. In the learning session, a total amount of 1.6 × 10^8^ steps of self-exploration were implemented to drive the learning and self-organization processes of the two neural networks. Other procedures were all identical to the first experiment.

#### 5.4.3. Tests and Results

In the test session, we used the noisy targets group that we used in the first experiment. Figure 14C shows the contributions of the posture and the direction controllers. Compared to the original wrist position, as shown by the green line in Figures 12, 14C, both groups show strong learning effect as the median final reaching errors after five motion steps are strongly reduced. The mean distance from the wrist to the targets during the entire test session was 59 cm, and the mean error of the reaching after first step driven by PTN was 6.21 cm. This is even smaller than the error from the same condition that was tested in the last experiment, which is 8.86 cm. The DTN's contribution afterward reduces the mean error to 3.19 cm. This is also better than the mean error in the last experiment, which was 3.28 cm. The difference between the two groups of errors is also significant [*F*_(1,98)_ = 7.77, *p* < 0.01].

The performance of the model in this test is generally better than the performance found in the first experiment even though we reduced the resolution in the joints from 6 to 25°. On the other hand, we expanded the range of each joint to 180°, which may explain the improvement in the performance. Moreover, the eye motion improves the contribution of the PTN, resulting in a decrease in the error.

## 6. Conclusions

In this paper, we investigated a model reproducing sensorimotor activities observed in human cognitive development. The model learns to coordinate sensory map representations with motor vector representations thereby generating accurate goal-directed reaching movements. We show that the implementation of the cyclopean map successfully provides the visual information in the guidance of reaching behaviors.

The experimental results with our proposed system show that its contrast-sensitive visual processing is able to locate an object's spatial center. In particular, a contrast balance method, the Ding-Sperling model, improves the object localization in the situation where two objects are spatially close to each other. The experimental results also show a good reaching performance measured by the Cartesian error between the end-effector and the target. With proper amount of learning, the model successfully contacts the target in almost half trials that have been tested, and the errors are within 5 cm in three quarters of the trials (condition “n3/3”). The two neural-networks, PTN and DTN, show their distinct and significant contributions during the test session. We also found our model robust in the noisy condition. Even though the median Cartesian error increases when noise is applied, there is no significant difference in the Cartesian error between “noisy” and “clear” conditions.

At present, our model is able to locate and reach the target in a relative low resolution. The model is not optimized according to the serial computing architectures and principles used in today's computing technology. In contrast, the model is built according to the massively-parallel computing-principle used in the nervous system. Thus, current computing technology limits the implementation of the model and we had to restrict the resolution of internal representations (i.e., number of neurons and layers) to be able to run our simulations in a reasonable time. Massively parallel analog computers can provide a much better platform for implementing our model. In terms of comparing serial computing technology with massively parallel neurocomputing, it suffices to highlight that even though neurons operate orders of magnitude slower than integrated circuits (time-scale of milliseconds vs. nanoseconds), for many sensory and motor tasks the brain outperforms computers both in accuracy and speed.

As mentioned in the introduction, an alternative approach to sensorimotor coordination could be based on the popular deep-learning methodology (Vos and Scheepstra, 1993; Takemura et al., 2018). Typically, a multi-layer feed-forward neural-network is set and initialized by random weight values. A training set is used, where the inputs represent the visual image or coordinates of the target whereas the outputs consist of joint angles. By using supervised learning, the error between the actual joint-angles and the desired joint-angles can be back-propagated to adjust the synaptic weights. Our approach is different in that it is (i) autonomous, (ii) unsupervised, and (iii) local. Unlike the aforementioned deep-learning approach, we do not need an external teacher who will generate training data and feed the training data to the network. Through circular reactions, our model autonomously generates its own training trials and data. The sensorimotor closed-loop in action automatically provides error signals in real-time. Hence, no supervision is needed. Finally, we embed explicitly map and vector representations in our model based on the neurophysiology of the primate brain, as opposed to starting “tabula rasa,” i.e., a network with randomly selected weights. These map representations and their coordinations are inspired by the organization and development of sensory systems in biology. As mentioned in the Biological Evidence Section, the explicit map representations in the model allow local learning. Learning is restricted to “sensitivity zones,” which represent local subsets of space. According to this approach, highly nonlinear relationships across the entire visual space can be simplified by local approximations, resulting in a much simpler learning approach.

## Data Availability Statement

The datasets presented in this study can be found in online repositories. The names of the repository/repositories and accession number(s) can be found below: https://github.com/hedch/reaching_project.

## Author Contributions

DH and HO conceived the study and reviewed, interpreted, and discussed the results. DH designed and implemented the simulations and generated the results and wrote the first draft of the manuscript. HO revised the manuscript. Both authors contributed to the article and approved the submitted version.

## Conflict of Interest

The authors declare that the research was conducted in the absence of any commercial or financial relationships that could be construed as a potential conflict of interest.

## Publisher's Note

All claims expressed in this article are solely those of the authors and do not necessarily represent those of their affiliated organizations, or those of the publisher, the editors and the reviewers. Any product that may be evaluated in this article, or claim that may be made by its manufacturer, is not guaranteed or endorsed by the publisher.

## Footnotes

^1^Traditionally, the term “allocentric” is used in this context. However, the prefix “allo” means “other” and does not explicitly state the property of being outside of the observer and does not highlight this contrast. Hence we use the term exocentric to contrast it with egocentric and to convey more directly its meaning.
